# Bridging the Neurosurgical Training Divide: Why Traditional Residency Models Are Obsolete for the Neurosurgeon of 2035

**DOI:** 10.7759/cureus.111511

**Published:** 2026-06-25

**Authors:** Faiqa I Khan, Muhammad Fawad Ul Hassan, Muhammad Sarwar, Haseeb Mehmood Qadri

**Affiliations:** 1 Neurological Surgery, Punjab Institute of Neurosciences, Lahore, PAK; 2 Neurological Surgery, National Hospital and Medical Center, Lahore, PAK; 3 General Surgery, Lahore General Hospital, Lahore, PAK

**Keywords:** automation, developing countries, medical education, neurosurgery, orthodox training, patient care, residency

## Abstract

The global neurosurgical educational framework is not equipped to address the disparity in demand globally. However, the demand for neurosurgical care is considerable. There is a substantial contrast between training programs in lower-middle-income countries (LMICs) and higher-income countries (HICs), each with its own limitations. Traditional time-based training models are now less effective. One way to mitigate this educational gap is by utilizing digital resources. Rather, a competency-based training model, supported by innovative technologies, is of paramount importance in this era.

## Editorial

Every year globally, 13.8 million individuals require neurosurgical care, and an estimated 23,000 additional neurosurgeons are required to bridge this gap [1]. The WHO recommends a ratio of 1 neurosurgeon per 200,000 people, and this ratio is severely unmet in LMICs, where there is often 1 neurosurgeon per 807,000 people [2]. Moreover, access to neurosurgical care is often delayed by more than four hours after a traumatic event [2]. The dilemma does not end with the workforce alone; there is also a lack of dedicated educational infrastructure and a standardized training curriculum. A notable example of this includes the lack of dissection tables and surgical skills labs in 70% of LMIC settings [1]. Furthermore, Villanueva PJ et al., in a global survey, reported a lack of training equipment, such as microscopes, and competency-building infrastructure in 35% of institutes [3]. Consequently, residents in LMICs spend their time on logistics and administrative work in addition to clinical learning [1]. Furthermore, in centers that are equipped with better facilities, the cost is skyrocketing. Families are sometimes obliged to bear costs up to 19.7 times their annual income to meet the expenses of a simple neurosurgical procedure [2]. Most of these centers are concentrated in urban settings, making care nearly unaffordable for rural families, falling short of the “within two-hour reach” recommendation of the Lancet Commission on Global Surgery, and leading trainees to prefer urban settings [4]. This eventually promotes brain drain in LMICs, and given these circumstances, trainees tend to opt for better training programs in developed countries and avoid returning due to operational shortages and capacity limitations [4].

In contrast to LMICs, HICs have problems centered on training disparities, since HICs dominate neurosurgical facilities despite having a low disease burden per consultant, such as 1 per 13,000 in Japan [4]. While the traditional paradigm has served medicine well, its assumption that “time equals competence and exposure guarantees proficiency” is becoming increasingly difficult to justify in surgical specialties. Clinical competencies and training of neurosurgeons have previously been measured primarily by case volumes, assuming that more cases seen equals better skills [5]. In fact, a 2022 survey by Sarpong K et al. concluded that there was no statistically significant difference in the availability of skills, number of procedures, and research opportunities between HICs and LMICs. Rather, there was a significant difference in weekly hour limits (p = 0.016) and subspecialty training (p = 0.001) [1]. The traditional training system seems to be time-bound rather than competency-based, with less personalization, which is a key element of a training program in promoting self-directed learning [5]. However, modern studies focus on competency-based training that depicts actual ability rather than experience count. It aims to replace case-number obsession and ensure quality training [5]. Moreover, across both Milestones 1.0 and 2.0, implemented by the Accreditation Council for Graduate Medical Education in neurosurgery, patient care remained the lowest-scoring competency [6].

Given these circumstances, reputable international neurosurgical societies such as the World Federation of Neurosurgical Societies (WFNS) have launched online webinars, in-house sessions, and online libraries to bridge the knowledge-based part of the gap. However, these collaborative models aim to address educational shortcomings magnified by economic challenges. A bibliometric analysis showed that a few LMICs, notably India, have tried to develop indigenous models to address the training gap [7]. Others have focused on “feasibility” or education. Thematic searches from HICs were more patient-focused, with terms such as “simulations,” “reconstruction,” or “segmentation” [7]. This shift reflects national-level imperatives, depicting the cumulative direction of the training model.

Digital apps and mobile resources are now proving beneficial in disseminating knowledge from global experts and improving the hands-on learning habits of trainees [8]. Neuroanatomy is the cornerstone of neurosurgery, and efforts have been made to make it accessible via digital platforms or simple mobile applications. Notable examples include The Rhoton Collection, UpSurgeOn, The Neurosurgical Atlas, NeuroSurgical.TV, and Brain Book [8]. Furthermore, neurosurgery demands minimal margin for error, and surgery is high-stakes. The workforce is inadequate to address global neurosurgical demand, and training skilled individuals is pivotal to bridging the gap. Davids J et al., in a meta-analysis, reported the use of high-fidelity simulators in neurosurgical skills training [9]. They showed a significant improvement in procedural knowledge (p = 0.001), accuracy (p < 0.001), speed (3.95 times faster), and depth perception [9]. However, there was no difference in procedure-related discomfort. In comparison, virtual/augmented reality or 3D-printed simulator technologies worked better, with greater realistic precision than cadaveric models [9]. Nonetheless, financial constraints in LMICs can challenge the acquisition of this equipment, coupled with ethical-legal concerns and skill shortages [9].

LMIC training is limited from logistical and operational viewpoints. Standalone capital movement and infrastructure building do not exclusively address global neurosurgical disparities. Non-technical skills, especially patient care, remain a predicament. In addition, the time-based training model is exhibiting obsolescence. While exposure is vital, competency-based tutoring via next-generation digital and analogue models is indispensable in the modern era of automation. Teaching merely through traditional methods is insufficient in the Industrial 4.0 revolution epoch for the neurosurgeon of 2035.
